# Differential response to topical lubrication in patient with dry eye disease, based on age

**DOI:** 10.1186/s12886-022-02609-2

**Published:** 2022-10-05

**Authors:** Yingxin Chen, Yajun Wu, Minghong Gao, Ruiyao Gao, Kai Zhang

**Affiliations:** Department of ophthalmology, the General Hospital of Northern Theater Command, No.83, Wenhua Road, Shenhe District, 110840 Shenyang, China

**Keywords:** Dry eye, Age, Ocular surface disease index score, Schirmer I test, Fluorescein break up time, Corneal fluorescence staining score

## Abstract

**Background:**

To compare the Ocular surface disease index (OSDI) score, Schirmer I test (SIT), fluorescein break up time (FBUT) and fluorescence staining (FLCS) score of dry eye patients at different ages.

**Methods:**

90 eyes of 90 patients with mild to moderate dry eye from September 2020 to September 2021 were retrospectively included and were divided into young group (20–39 years, n = 29), middle-age group (40–59 years, n = 30), and elder group (> 60 years, n = 31). Patients were given a 28-day topical lubricating ocular surface and repair-promoting drugs combined with local physical therapy. Patients were followed up at 7, 14 and 28 days. The OSDI score, SIT, FBUT and FLCS score were examined.

**Results:**

There were differences between the OSDI score in three groups at each time point (all P < 0.001). SIT were different among the three groups (F = 350.61, P < 0.001), and a time effect was found (F = 80.87, P < 0.001). SIT at 14 and 28 days after treatment in middle-age and elder groups were lower than young group (all P < 0.001). SIT at 7, 14 and 28 days in elder group were lower than middle-age group (all P < 0.001). FLCS score was lower at 28 days than other time points (all P < 0.001).

**Conclusion:**

Dry eye patients are given a 28-day topical lubricating ocular surface and repair-promoting drugs combined with local physical therapy, which can promote tear secretion, film stability, and the recovery of corneal integrity. Age affects the treatment effect of mild to moderate dry eye, among which tear secretion is the most significant.

## Background

Dry eye is now considered as an ocular surface disease characterized by loss of homeostasis in the tear film, accompanied by ocular symptoms, tear film instability and hyperosmolarity, inflammation and damage to the ocular surface [1].The maintenance of the normal state of the ocular surface is inseparable from the coordinated regulation of various parts of the ocular surface tissue, thereby maintaining a healthy state of tears and tear film. Any damage to any link will affect the integrity of the tear film, and even affect its function, leading to the appearance of dry eye [2, 3].

Several approaches including ocular surface disease index (OSDI) score, Schirmer I test (SIT), fluorescein break up time (FBUT) and corneal fluorescence staining (FLCS) score [2, 4]. Studies have shown that dry eye is an age-related degenerative disease that is expected to cause an increasing public health burden worldwide as the population ages [5–7]. However, the changes in symptoms and signs of dry eye patients of different ages before and after treatment and whether there are differences need to be reported by more studies [8]. The Tear Film and Ocular Surface Society (TFOS) Dry Eye Workshop II (DEWS II) Epidemiological Report provides evidence of gender differences in the signs of dry eye with age, but results are variable and systematic stratified studies are advocated [5]. Therefore in this study, patients were divided into three groups according to age, and we aimed to compare the OSDI score, SIT, FBUT and FLCS score of dry eye patients at different ages before and after treatment.

## Methods

### Patients

A total of 90 eyes of 90 patients with mild to moderate dry eye who visited our outpatient clinic from September 2020 to September 2021 were retrospectively included. All patients provided written informed consent, and this study protocol was performed in accordance with the Declaration of Helsinki reviewed and was approved by the Ethics Committee of General Hospital of Northern Theater Command (2022-027). All patients had completed fundus examinations and slit lamp examinations to exclude other ocular surface diseases and fundus diseases except dry eye.

Inclusion criteria were patients (1) with symptoms including blurred vision, dry eyes, and foreign body sensation, which can be diagnosed as mild to moderate dry eye; (2) with no other dry eye related treatment has been received within 1 month; and (3) aged from 18 to 78 years, and do not suffer from mental and psychological diseases. Exclusion criteria were patients (1) with other ophthalmic diseases who need local or systemic use of other drugs that may affect the secretion of tears; (2) who had previous chemical burns, eye surgery, or contact lenses; (3) with eyelid insufficiency, conjunctival sac relaxation, ectropion, or blepharospasm that affect the normal metabolism of drugs; (4) who were pregnant and lactating women or those who were taking hormone-related drugs; (6) those with immune-related diseases such as Sjögren’s syndrome, Stevens-Johnson syndrome, abnormal thyroid function, and other serious diseases such as tumor diseases.

One eye of each patient was selected for the study, and if both eyes of the patient met the inclusion criteria, the right eye was selected as the study eye. The patients were divided into three groups according to age. Patients in young group were 20–39 years old, with a total of 29 cases and 29 eyes; Patients in middle-age group were 40–59 years old, with a total of 30 cases and 30 eyes; Patients in elder group were aged 60 years and above, with a total of 31 cases and 31 eyes. All patients were examined by the same ophthalmologist to complete their OSDI score, SIT, FBUT and corneal FLCS score at day 0.

### Therapeutic method

All patients were given meibomian gland massage once a week for 4 times in a row by the same ophthalmic nurse. The therapeutic method was reported previously [9].

In addition, patients in each group were given polyethylene glycol eye drops (Siran, Alcon Laboratories, Inc) and vitamin A palmitate ophthalmic gel (Ziyang, Shenyang Xingqi Pharmaceutical Co., Ltd.) 3 times a day for 28 days. After each massage, levofloxacin eye drops (Colobito, Santian Pharmaceutical Co., Ltd.) were applied to the affected eye 3 times a day and gatifloxacin Ophthalmic gel (Diyou, Shenyang Xingqi Ophthalmic Co., Ltd.) was applied to the affected eye once a night. These two drugs were discontinued after continuous use for 3 days. Eye drops were performed by the same physician according to the standard procedure. Patients were followed up 7 days, 14 days and 28 days after treatment.

### Observational index

All patients were examined by the same ophthalmologist to complete their OSDI score, SIT, FBUT and corneal FLCS score. The OSDI score include the scores of all 12 completed questions, ranging from 0 to 100 [10]. For SIT, take a 5 mm×35 mm standard filter paper strip (Tianjin Jingming New Technology Development Co., Ltd.), one end is folded for 5 mm, and the other end hangs down naturally and was inserted it into the junction of the middle and outer 1/3 of the lower eyelid. The filter paper was removed after 5 min, and the wetted length of the filter paper at the folded position < 10 mm can indicate the dry eye [11].

For FBUT, after soaked with chloramphenicol eye drops (Changchun Dirui Pharmaceutical Co., Ltd.), the fluorescein test strip (Tianjin Jingming New Technology Development Co., Ltd.) was then applied to the patient’s lower eyelid conjunctival sac. The patient blinked several times and stared forward, the cobalt blue light of the slit lamp microscope was selected for observation, and the stopwatch was used for measurement. The time from the patient opened his eyes after the last blink until the first randomly distributed dry spot on the cornea occurred was recorded for 3 consecutive times. An average time is less than 10s can indicate the dry eye [11].

For FLCS, a drop of chloramphenicol eye drops was used to infiltrate the tip of the fluorescein test strip and the strip was then placed in 1/3 of the patient’s lower eyelid conjunctival sac. After blinking 3–4 times, the cornea was observed under the cobalt blue light of the slit lamp to see if the cornea was stained, and the score was 12 points: the cornea is divided into 4 quadrants, with each quadrant of 0–3 points; 0 points for no staining, 1 point for 1–5 punctate staining, 2 points for 5–30 spotty staining but unfused staining, and 3 points for corneal punctate staining fusion, filaments, and ulcers [12].

### Statistical analysis

Statistical analysis was performed using SPSS version 23.0 (IBM Corp.). Values are presented as the mean ± standard deviation or numbers (percentage). One-way analysis of variance (ANOVA) was used for multiple continuous variables and the chi-square test was used to compare categorical data among the groups. Significance in OSDI score, SIT, FBUT and FLCS score were tested with two-way ANOVA followed by Bonferroni test. P < 0.05 was considered to indicate a statistically significant difference.

## Results

There were no significant differences in gender, dry eye duration, diabetes history, smoking history, OSDI score, SIT, FBUT and FLCS score before treatment among the three groups (P > 0.05) (Table 1).

**Table 1:** Basic information

Variables	Young group (n = 29)	Middle-age group (n = 30)	Elder group (n = 31)	P value
Male, n(%)	14(48.28)	14(46.67)	16(51.61)	0.925
Dry eye duration (month) (means ± SD)	4.37 ± 2.73	4.28 ± 2.30	4.56 ± 2.80	0.912
Diabetes history, n(%)	5(17.24)	8(26.67)	7(22.58)	0.683
Smoking history, n(%)	8(27.59)	6(20)	6(19.35)	0.783
OSDI score(means ± SD)	26.00 ± 5.47	26.10 ± 5.55	25.84 ± 5.24	0.979
SIT (mm/5min) (means ± SD)	4.59 ± 1.24	4.50 ± 1.17	4.42 ± 1.29	0.872
FBUT (s) (means ± SD)	4.31 ± 1.34	4.13 ± 1.55	4.00 ± 1.39	0.682
FLCS score (means ± SD)	3.10 ± 1.94	3.23 ± 1.87	3.45 ± 1.71	0.794

OSDI, ocular surface disease index; SIT, Schirmer I test; FBUT, fluorescein break up time; FLCS, corneal fluorescence staining

As shown in Table 2, OSDI score was not statistically different among the groups (F = 3.22, P = 0.06), and a significant time effect was found (F = 427.21, P < 0.001). There was also a significant interaction between time and groups (F = 7.01, P < 0.001). Regarding within-group differences, there were statistically significant differences between the three groups at each time point before and after treatment (all P < 0.001).

**Table 2:** OSDI score among three groups

Variables (means ± SD)	Young group (n = 29)	Middle-age group (n = 30)	Elder group (n = 31)	Mixed ANONA (P value)		
				Group effect	Time effect	Interaction effect
Before treatment	26.07 ± 5.47	26.10 ± 5.55	25.84 ± 5.24	0.057	< 0.001	< 0.001
7 days after treatment	23.31 ± 4.98^*^	20.30 ± 4.47^*^	21.32 ± 4.20^*^			
14 days after treatment	19.10 ± 4.14^*#^	15.97 ± 3.82^*#^	17.74 ± 3.68^*#^			
28 days after treatment	14.97 ± 3.91^*#&^	10.67 ± 2.92^*#&^	13.26 ± 3.74^*#&^			

OSDI, ocular surface disease index

^*^P < 0.05, compared with OSDI score before treatment

^#^P < 0.05, compared with OSDI score 7 days after treatment

^&^P < 0.05, compared with OSDI score 14 days after treatment

SIT was statistically different among the three groups (F = 350.61, P < 0.001), and a significant time effect was also found (F = 80.87, P < 0.001). There was a significant interaction between time and groups (F = 10.70, P < 0.001). SIT at 14 and 28 days after treatment in both middle-age group and elder group were lower than young group (all P < 0.001). SIT at 7, 14 and 28 days after treatment in elder group were lower than middle-age group (all P < 0.001). Regarding within-group differences, there were statistically significant differences between the three groups at each time point before and after treatment (all P < 0.001)(Table 3).

**Table 3:** SIT among three groups

Variables (means ± SD)	Young group (n = 29)	Middle-age group (n = 30)	Elder group (n = 31)	Mixed ANONA (P value)		
				Group effect	Time effect	Interaction effect
Before treatment	4.59 ± 1.24	4.50 ± 1.17	4.42 ± 1.29	< 0.001	< 0.001	< 0.001
7 days after treatment	6.55 ± 1.40^*^	6.10 ± 1.52^*^	5.10 ± 1.51^ab*^			
14 days after treatment	8.41 ± 1.94^*#^	7.40 ± 1.83^a*#^	6.23 ± 1.56^ab*#^			
28 days after treatment	9.72 ± 1.77^*#&^	8.10 ± 1.45^a*#&^	6.97 ± 1.45^ab*#&^			

SIT, Schirmer I test

^*^P < 0.05, compared with SIT before treatment

^#^P < 0.05, compared with SIT 7 days after treatment

^&^P < 0.05, compared with SIT 14 days after treatment

^a^P<0.05, compared with young group

^b^P<0.05, compared with middle-age group

As shown in Table 4, FUBT was not statistically different among the groups (F = 2.66, P = 0.08), and a significant time effect was found (F = 56.63, P < 0.001). There was also a significant interaction between time and groups (F = 4.58, P < 0.001). No significant differences in FUBT at 28 days after treatment were found compared with FUBT at 14 days after treatment in both middle-age group and elder group. In addition, there were statistically significant differences between the three groups at other time point before and after treatment (all P < 0.001).

**Table 4:** FUBT among three groups

Variables (means ± SD)	Young group (n = 29)	Middle-age group (n = 30)	Elder group (n = 31)	Mixed ANONA (P value)		
				Group effect	Time effect	Interaction effect
Before treatment	4.31 ± 1.34	4.13 ± 1.55	4.00 ± 1.39	0.076	< 0.001	< 0.001
7 days after treatment	5.21 ± 1.61^*^	4.83 ± 1.68^*^	4.42 ± 2.01^*^			
14 days after treatment	5.86 ± 1.87^*#^	5.33 ± 1.69^*#^	5.00 ± 1.93^*#^			
28 days after treatment	6.93 ± 1.83^*#&^	5.73 ± 2.02^*#^	5.13 ± 1.98^*#^			

FBUT, fluorescein break up time

^*^P < 0.05, compared with FBUT before treatment

^#^P < 0.05, compared with FBUT 7 days after treatment

^&^P < 0.05, compared with FBUT 14 days after treatment

As shown in Table 5, FLCS score was not statistically different among the groups (F = 1.23, P = 0.30), and a significant time effect was found (F = 49.625, P < 0.001). There was no significant interaction between time and groups (F = 1.533, P = 0.170). Regarding within-group differences, FLCS score was lower at 28 days after treatment than that before treatment and 7 and 14 days after treatment(all P < 0.001).

**Table 5:** FLCS score among three groups

Variables (means ± SD)	Young group (n = 29)	Middle-age group (n = 30)	Elder group (n = 31)	Mixed ANONA (P value)		
				Group effect	Time effect	Interaction effect
Before treatment	3.1 ± 1.94	3.23 ± 1.87	3.45 ± 1.71	0.299	< 0.001	0.170
7 days after treatment	2.76 ± 1.90	3.07 ± 1.43	3.13 ± 1.78^*^			
14 days after treatment	2.41 ± 1.78^*^	2.73 ± 1.78^*#^	2.94 ± 1.71^*^			
28 days after treatment	1.10 ± 0.82^*#&^	1.70 ± 1.32^*#&^	2.32 ± 1.50^*#&^			

FLCS, corneal fluorescence staining

^*^P < 0.05, compared with FLCS score before treatment

^#^P < 0.05, compared with FLCS score 7 days after treatment

^&^P < 0.05, compared with FLCS score 14 days after treatment

## Discussion

According to recent epidemiological studies, the prevalence of dry eye has increased significantly, and there is a positive correlation between increasing age and clinical signs, clinical markers of dry eye, aqueous tear deficiency, and meibomian gland dysfunction [13, 14]. Therefore, dry eye is considered a multifactorial, age-related degenerative disease that progresses with cumulative lifetime exposure to multiple environmental and physiological factors, leading to hormonal modulation, neurosensory pathways, ocular Changes in inflammation and tear film homeostasis [15, 16]. As the number of elderly people will continue to increase in the future, and the life expectancy of human beings will continue to increase, which will lead to the larger proportion of patients with dry eyes. Therefore, we need to further understand the age-related dry eye in detail, so as to improve the status quo and guide treatment [17].

OSDI scores, SIT, FBUT, and FLCS scores of the OSDI scores in three groups at 28 days after treatment were significantly improved compared with those before treatment and 7 days after treatment, suggesting that long term treatment of dry eyes provides better results.

There were statistically significant differences between the three groups at each time point before and after treatment. Overall, though not significantly different, the OSDI scores of middle-age group at each time point were lower than the other two groups, considering that the cumulative exposure due to advances in science and technology and lifestyle factors may vary by age group [18]. In addition, ocular nerve sensitivity and response sensitivity decreases with increasing age.

SIT is the most direct way to detect tear secretion, and the increase of tear secretion can directly improve the symptoms of ocular surface discomfort and visual quality of patients. SIT at 7, 14 and 28 days after treatment in elder group were lower than middle-age group in this study. The recovery of tear deficiency is more pronounced and faster in patients under 60 years of age than in those over 60 years of age [18]. We believe that the reason is that sex hormone levels may regulate tear production through their effects on the ocular surface to stabilize the ocular surface environment, and with age, hormone levels gradually decline, affecting tear production to a certain extent [19, 20]. Hormone replacement therapy appears to have a beneficial effect on lacrimal secretion, based on evaluation of the tear secretion test, and this effect is age-related [21]. With regard to age-based differences in the clinical efficacy of dry eye, in the case of SIT, tear secretory glands and their neural connections comprise tear functional units, and abnormalities in some of these units can lead to dysfunction. The normal lacrimal gland is composed of 80% acinar cells, which store and secrete tear components. An animal study found that age causes progressive changes in lacrimal acinar cells, and the type of acinar changes from initially serous to serous-mucinous acinus, and then gradually to mucinous acinus [22]. In addition, with increasing age, the lacrimal gland develops excessive structural damage, mast cell infiltration, periductal fibrosis, acinar atrophy, and chronic inflammation. The ability of the acini to synthesize and secrete proteins gradually decreased or disappeared in mice aged 3 months to 5 months to 20 months to 24 months [23]. These morphological and secretory changes also explain the decrease in tear secretion with age.

FBUT is a method for evaluating the stability of the tear film of patients, which is reproducible, less irritating to the patient, and the results are objective and accurate. Therefore, it is widely used in clinical practice. In this study, the FBUT measurement of all patients was completed by the same physician in the same environment, which eliminated the influence of external factors such as the size of the eye cleft, the environmental humidity, and the dose of fluorescein staining. Though not significantly different, FUBT at 28 days after treatment of young group were higher than the other two groups. The optimal prognostic cut-off age for tear film instability and hyperosmolarity may occur in the 40th year of life, between 33 and 38 years old. In elderly patients with dry eye, high osmotic pressure may have an association between and lower FBUT [18], and the recovery of tear film stability is more pronounced and faster in patients under 40 years of age than in those over 40 years.

Corneal FLCS is a commonly used clinical method for evaluating corneal damage. Corneal and conjunctival staining of the ocular surface is considered to be a sign of a more severe stage in the later development of dry eye [14, 18, 24], indicating compromised corneal, conjunctival, and lid margin epithelial integrity [25, 26]. FLCS at 28 days after treatment in three groups were significantly lower than other time points. Changes in tear film homeostasis in dry eyes may increase the drying risk due to tear film instability and excessive evaporation through an inflammatory cascade triggered by tear film hyperosmolarity, resulting in damage to the ocular surface epithelium and blinking with reduced lubrication and increased friction [16, 27, 28], the delayed onset of this injury therefore requires long-term treatment [29].

The sample size and short follow-up time were limitations of this study. In addition, this study is lack of relevant biochemical indicators. The lack of significant difference in baseline OSDI among different groups is also a limitation to the study. Blepharitis in patients were not assessed and studied as a possible co-founding variable in this study. A higher prevalence of dry eye in females were not shown in our study, which may be due to the small sample size. Therefore, large-scale and long-term researches remain to be done in the future. During the study period, all patients in three age groups wore masks due to the 2019 novel coronavirus (COVID-19). However “Mask associated dry eye (MADE)”, as a known entity, was not discussed in detail.

## Conclusion

Dry eye patients are given a 28-day topical lubricating ocular surface and repair-promoting drugs combined with local physical therapy, which can relieve symptoms, promote tear secretion, improve tear film stability, and promote the recovery of corneal and ocular surface integrity. As a long-term, chronic ocular surface disease, durable treatment is required. Age affects the treatment effect of patients with mild to moderate dry eye, among which the tear secretion is the most significant. Treatment of dry eye should be detected and managed early on to protect the ocular surface.

## Data Availability

All data generated or analyzed during this study are included in this article.

## Abbreviations

OSDI: Ocular surface disease index
SIT: Schirmer I test
FBUT: Fluorescein break up time
FLCS: Fluorescence staining
TFOS: Tear Film and Ocular Surface Society
DEWS II: Dry Eye Workshop II
ANOVA: Analysis of variance
